# Direct Visualization of Surface Structure and Charge States of Ceria‐Supported Gold Catalysts Under Redox Conditions

**DOI:** 10.1002/advs.202508554

**Published:** 2025-07-10

**Authors:** Ryotaro Aso, Takehiro Tamaoka, Hideto Yoshida, Hajime Hojo, Hiroki Sano, Yoshihiro Midoh, Hisahiro Einaga, Toshiaki Tanigaki, Yasukazu Murakami

**Affiliations:** ^1^ Department of Applied Quantum Physics and Nuclear Engineering Kyushu University 744 Motooka, Nishi‐ku Fukuoka 819‐0395 Japan; ^2^ The Ultramicroscopy Research Center Kyushu University 744 Motooka, Nishi‐ku Fukuoka 819‐0395 Japan; ^3^ SANKEN The University of Osaka 8‐1 Mihogaoka, Ibaraki Osaka 567‐0047 Japan; ^4^ Department of Advanced Materials Science and Engineering, Faculty of Engineering Sciences Kyushu University 6‐1 Kasuga‐koen Kasuga Fukuoka 816‐8580 Japan; ^5^ Graduate School of Information Science and Technology The University of Osaka 1‐5 Yamadaoka Suita Osaka 565‐0871 Japan; ^6^ Research and Development Group Hitachi, Ltd. 2520 Hatoyama Saitama 350‐0395 Japan; ^7^ Present address: Morphological Research Laboratory Toray Research Center Inc. 3‐2‐11, Sonoyama Otsu Shiga 520‐8567 Japan

**Keywords:** charge states, electron holography, environmental TEM, supported gold nanoparticle catalysts, surface structures

## Abstract

Observing the surface structure and charge dynamics of catalysts during catalytic reactions is crucial for elucidating reaction mechanisms. However, nanoscale characterization of the catalyst structure and charge states in the presence of reactive gases presents experimental challenges. Here, the structures and charge states of a gold nanoparticle (NP) are directly visualized on ceria during redox cycles using electron holography, a method related to transmission electron microscopy. The introduction of oxidizing O_2_ gas to the microscope led to structural changes on the NP surface and decrease the intrinsic negative charge of the NP. Conversely, under reducing H_2_ gas, the surface structure and charge state of the NP remained almost unchanged compared to those in vacuum. Systematic analysis revealed that the injection and removal of O_2_ gas caused reversible changes in the charge state of the NP within the range of a few electrons. The effect of O_2_ gas on charging of the NP is confirmed by first‐principles calculations. These findings demonstrate the potential of electron holography in gas environments for advancing the understanding the reaction mechanisms on heterogeneous catalysts.

## Graphical abstract

In the characterization of supported metal catalysts, a crucial aim is to conduct nanoscale analysis of the surface structure and charge dynamics in reactive gases. This study uses high‐precision electron holography in gas environments to observe the charge states of Au nanoparticles (NPs) supported on CeO_2_ during redox cycles. It reveals that the injection and removal of O_2_ gas can cause reversible changes in the charge state of the NPs.

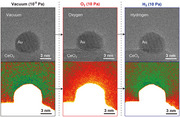

## 1 Introduction

Noble‐metal nanoparticles (NPs) dispersed onto and immobilized on supports serve as highly effective heterogeneous catalysts in various applications, including the chemical industry, automotive exhaust gas purification, gas sensors, and fuel cells.^[^ ^1^, ^2^ ^]^ Au, although catalytically inactive in its bulk form, exhibits substantial catalytic performance in nanostructures such as clusters, atomic layers, and NPs on supports, as well as sponge and tube forms.^[^ ^3^, ^4^, ^5^, ^6^, ^7^, ^8^, ^9^, ^10^ ^]^ Haruta et al. discovered that supported Au NPs exhibit catalytic activity toward the low‐temperature oxidation of CO.^[^ ^7^, ^8^, ^9^, ^11^ ^]^ Additionally, supported Au NPs are reportedly active in the water–gas shift reaction.^[^ ^10^ ^]^

The catalytic properties of such heterogeneous catalysts are heavily influenced not only by the element, size, and morphology of the metal NPs and supports but also by the junction characteristics of the metal–support interface.^[^ ^12^ ^]^ The strong metal–support interaction (SMSI),^[^ ^13^, ^14^ ^]^ which involves the migration of atoms across the interface between NPs and reducible oxide supports, strongly affects the morphology and activity of NP catalysts. Additionally, charge transfer across the interface, discussed in the framework of electronic metal–support interactions (EMSIs),^[^ ^15^, ^16^ ^]^ has attracted intensive attention because it influenced the charge states that contribute to catalytic activity. Therefore, interface engineering has emerged as a critical strategy for enhancing catalytic performance. Catalytic activity is governed by the surface properties of catalytic NPs and by the metal–support interface, which acts as an active site for reactions involving gas molecules.^[^ ^17^ ^]^ Thus, directly elucidating the changes in the structure and charge at the surface and interface of NP catalysts during catalytic reactions is essential for comprehensively understanding catalytic mechanisms.

For the analysis of the surface properties of nanomaterials, various spectroscopic^[^ ^17^, ^18^, ^19^, ^20^ ^]^ and probe microscopy^[^ ^21^, ^22^, ^23^, ^24^, ^25^, ^26^ ^]^ techniques are widely used to measure the charge states and work functions of metal surfaces and to identify active sites on catalysts, even under reaction conditions. By contrast, (scanning) transmission electron microscopy ((S)TEM) has been extensively used for atomic‐level structural analysis of catalysts.^[^ ^27^, ^28^, ^29^, ^30^ ^]^ Additionally, electron holography, a TEM‐based method, has been employed to elucidate the structures and charge states of nanomaterials simultaneously.^[^ ^31^, ^32^, ^33^, ^34^, ^35^, ^36^ ^]^ Recently, high‐precision electron holography has attained the simultaneous crystal‐structure analysis and charge measurement of Pt NPs supported on TiO_2_ (Pt/TiO_2_), demonstrating that both structural distortions in the NPs and slight charge transfer at the metal–support interface influence the charge state of the NPs.^[^ ^36^ ^]^ In nanoscale charge analysis, the novel technique of 4D STEM^[^ ^37^ ^]^ has revealed that the charge state of Au NPs on SrTiO_3_ varies depending on the chemical state differences of the support surface.^[^ ^38^ ^]^

A formidable challenge in electron microscopy involves in situ analysis of the structure and charge states of catalysts during catalytic reactions. Environmental transmission electron microscopy (ETEM) allows for atomic‐resolution observation of catalysts under gas atmospheres.^[^ ^39^, ^40^, ^41^, ^42^, ^43^, ^44^, ^45^ ^]^ Owing to advancements in electron microscopy technologies, such as the inventions of aberration correctors and direct detection cameras, ETEM has enabled tracking of structural changes at the atomic level in gas or liquid environments.^[^ ^45^, ^46^, ^47^ ^]^ Although structural changes, including SMSI, during catalytic reactions have been analyzed, charge measurements associated with EMSI still face substantial challenges. Electron holography in gas atmospheres has been attempted by using ETEM;^[^ ^48^, ^49^, ^50^ ^]^ however, charge measurements from NP catalysts in working conditions remain highly challenging due to several technical problems. One significant challenge is the dynamic interaction between gas molecules and catalyst surfaces, which introduces fluctuations that affect measurement accuracy. Additionally, electron‐beam‐induced charging and gas‐induced electron scattering introduce further uncertainties, complicating the reliable assessment of charge states and precise measurements. In the present study, using high‐precision electron holography combined with high‐resolution ETEM, we elucidated changes in the charge state of metal NP catalysts in the presence of reactive gases. By implementing advanced control over both the gas environment and electron irradiation, we conducted structural and charge‐state analyses of Au NPs on ceria (Au/CeO_2_) as a practical catalyst in the presence of O_2_ and H_2_ as representative oxidizing and reducing gases, respectively. Simultaneous visualization of the structures and charge states of practical catalysts with high spatial resolution and high sensitivity represents a significant advancement in understanding catalytic reaction mechanisms on heterogeneous catalysts.

## 2 Results and Discussion

### 2.1 Structural and Charge Visualization of a Supported Au NP in Gas

**Figure 1:**
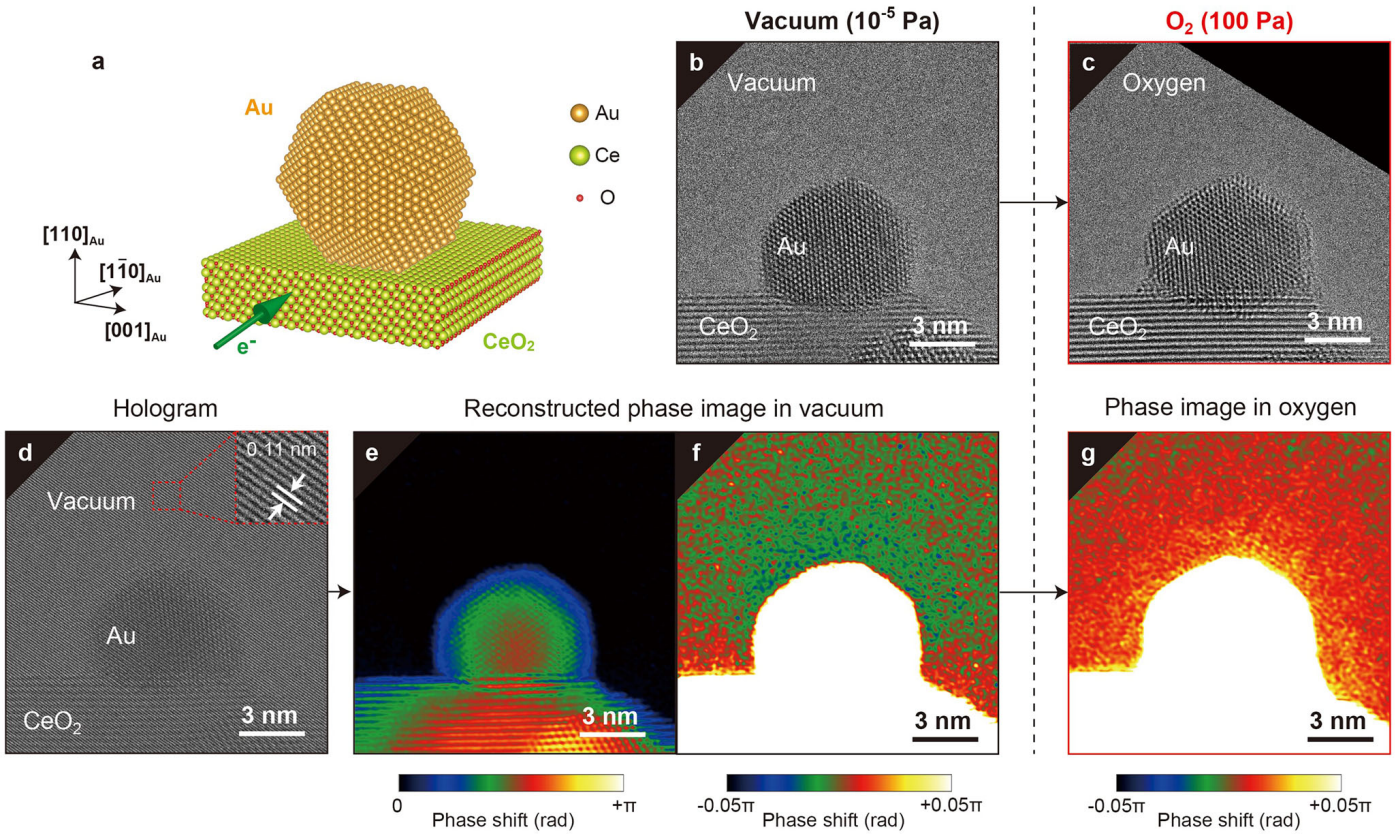
Structural and charge visualization of a supported NP in vacuum and gas. a) Schematic of the crystal structure of a Au NP supported on CeO_2_ (111), where the electron beam enters the specimen along the [1–10]_Au_ direction. b,c) TEM images of Au/CeO_2_ taken in (b) vacuum and (c) O_2_ (*P* = 100 Pa). d) Hologram image of Au/CeO_2_, where the inset shows the interference fringe pitch. e,f) Reconstructed and noise‐reduced phase images from (d), corresponding to (b). The image in (f) highlights the weak phase shift outside the Au NP. g) Reconstructed and noise‐reduced phase image after O_2_ gas insertion from (e), corresponding to (c).

The Au/CeO_2_ catalyst was prepared using the deposition–precipitation method^[^ ^51^ ^]^ (see Section S1, Supporting Information). The Au NPs, which exhibited particle sizes smaller than ∼10 nm, were dispersed and supported on CeO_2_ (Figure S1, Supporting Information). A single Au NP supported at the edge of CeO_2_ was selected for analysis. As shown in **Figure** **1**a, the electron beam used for observation was irradiated along the [1–10] direction of the face‐centered cubic (fcc) Au NP, which was approximately parallel to the Au/CeO_2_ interface. This observation direction is optimal for analyzing the NP and support crystal structures. In Figure 1b, the Au NP preserved in vacuum (i.e., reduced pressure of 10^−5^ Pa, attained by the electron microscope) exhibited a fully crystalline structure with an fcc lattice, where the Au (110) plane was parallel to the CeO_2_ (111) plane. When 100 Pa of O_2_ gas was introduced into the electron microscope (Figure 1c), the outermost atomic layers of the Au NP became disordered, as evidenced by a distinct image contrast compared to that of the original Au NP. A similar surface disordering of Au NPs in O_2_ gas has been reported,^[^ ^45^ ^]^ suggesting that the dissociation of oxygen molecules at the Au/CeO_2_ interface under electron irradiation can generate oxygen atoms or active oxygen species that diffuse on, adsorb onto, and desorb from the Au NP surface.^[^ ^42^ ^]^ Notably, the SMSI effect, such as in the case of TiO_2_‐coated Au NPs observed in Au/TiO_2_,^[^ ^44^ ^]^ is unlikely to occur with Au/CeO_2_ because the lattice framework of Ce atoms in CeO_2_ remains stable during the desorption and absorption of oxygen.^[^ ^42^ ^]^ Therefore, the disordering of the Au NP surface observed in the present study is likely to be caused by the reaction between Au and O_2_ gas.

To investigate the charge state of the Au NP supported on CeO_2_, we performed high‐precision electron holography.^[^ ^36^ ^]^ As explained elsewhere,^[^ ^52^ ^]^ electron holography reveals the electrostatic field by measuring the phase shift in the incident electron wave. The phase information is stored in an electron hologram which is made of interference fringes of electron waves. Figure 1d presents a typical electron hologram acquired in vacuum, showing clear interference fringes. To improve the accuracy of the phase analysis, noise reduction in the phase reconstruction of electron holograms was applied using a wavelet hidden Markov model (WHMM)^[^ ^53^ ^]^ (Section S3, Supporting Information). The reconstructed and noise‐reduced phase image in Figure 1e showed the phase change within the Au NP and CeO_2_. In electron holography studies, the phase shift observed in the region inside of Au NP originates mainly from the mean inner potential of the Au NP and dynamical electron scattering,^[^ ^36^, ^52^ ^]^ which obscure weak signals related to the NP charging. Conversely, the weak phase shift outside of the Au NP (Figure 1f) directly provides the charge information because the region outside of the NP is free from disturbance caused by the mean inner potential and the dynamical electron scattering. In the case of the Au NP in vacuum (i.e., without the introduction of gases, Figure 1f), the phase change outside the NP was negative (i.e., the phase decreased toward the NP), indicating that the Au NP was negatively charged. However, when 100 Pa of O_2_ gas was introduced (Figure 1g), the negative phase change outside the NP became positive (i.e., the phase increased toward the NP). These results indicate that the negative charge of the Au NP decreases and the NP becomes slightly positively charged due to the introduction of O_2_ gas.

### 2.2 Effect of Reactive Gases on Supported Au NP Catalysts

**Figure 2:**
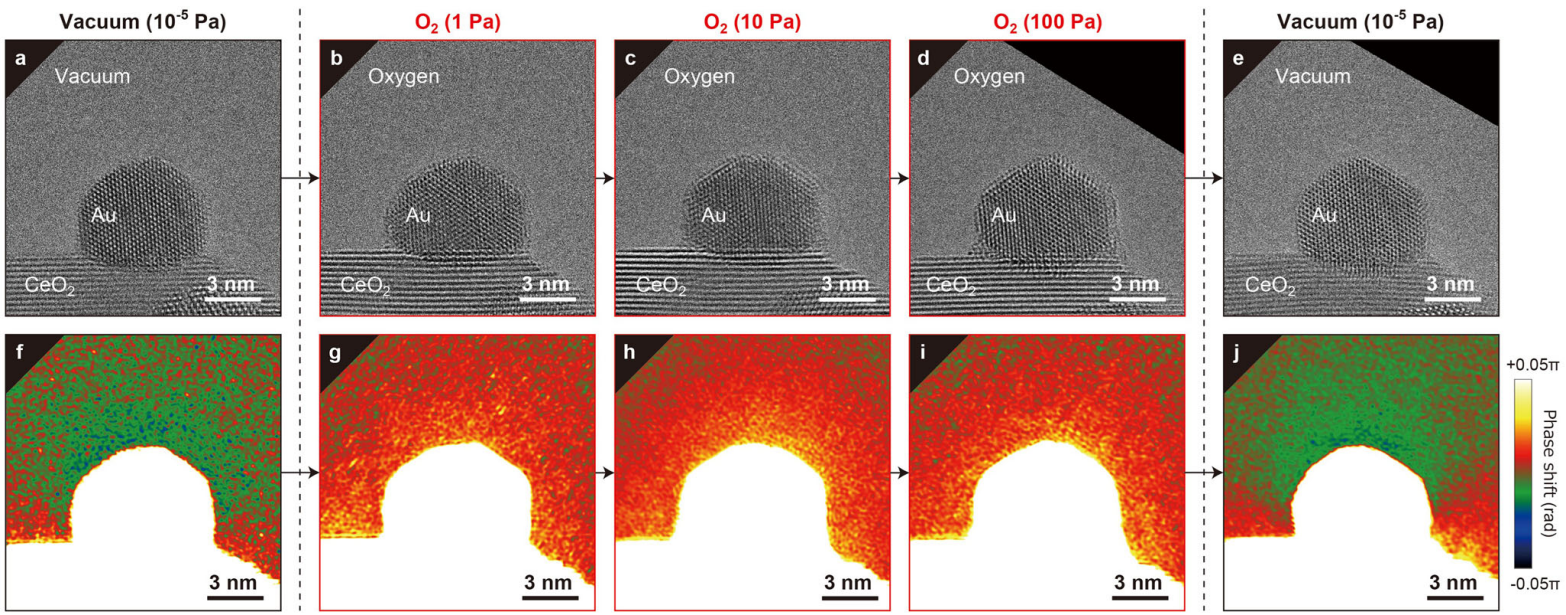
Structure and charge changes of Au/CeO_2_ accompanying O_2_ gas insertion and removal. a–d) TEM images of Au/CeO_2_ in (a) vacuum and (b) 1 Pa, (c) 10 Pa, and (d) 100 Pa of O_2_ gas. e) TEM image of Au/CeO_2_ in vacuum after O_2_ gas removal. f–j) Noise‐reduced phase images corresponding to (a–e), respectively.

To further examine the gas‐induced changes in the crystal structure and the charge state of the supported Au NP, we systematically varied the pressure of the introduced O_2_ gas up to 100 Pa. In contrast to the TEM image of the supported Au NP in vacuum (**Figure** **2**a, corresponding to Figure 1b), Figure 2b–d shows TEM images of the Au NP as the O_2_ gas pressure was increased to 1, 10, and 100 Pa, respectively. When 1 Pa of O_2_ gas was introduced (Figure 2b), the Au NP surface was slightly disordered. At O_2_ gas pressures of 10 Pa or higher (Figure 2c,d), the disordering of the Au surface became more pronounced. The ordered crystal planes on the Au surface were restored when the O_2_ gas was removed and the system was returned to vacuum (Figure 2e). Additionally, the lateral dimensions of the Au NP slightly expanded upon O_2_ gas injection, indicating that oxygen partial pressure plays a key role in morphological changes. A previous study has shown that Au NPs in an oxygen environment undergo structural transitions depending on the introduced oxygen partial pressure,^[^ ^42^ ^]^ with higher pressures favoring rounded structures and lower pressures leading to faceted morphologies. This highlights the dynamic nature of Au NP structural evolution under varying oxygen gas conditions.

The phase images corresponding to these TEM images are shown in Figure 2f–j. In vacuum, the supported Au NP was negatively charged (Figure 2f). When O_2_ gas was introduced (Figure 2g–i), the negative phase outside the Au NP decreased, shifting toward positive charge. When the O_2_ gas was removed and the system was returned to vacuum (Figure 2j), the phase change returned to negative, indicating restoration of the negative charge. To estimate the charge amount of the AuNP, curve fitting for the slight phase shift gradient outside of the NP, as revealed by high‐precision electron holography, was utilized (Figures S2 and S3, Supporting Information). This approach assumes that the Au NP is spherical and the total charge could be approximated as a point charge.^[^ ^36^ ^]^ The charge amount of the Au NP in vacuum could be deduced from Figure 2f to be −4 *q*_e_, on the order of the elementary charge, *q*_e_. When 1 Pa of O_2_ gas was introduced, the charge became +4 *q*_e_; even when the gas pressure was increased to 10 and 100 Pa, the charge amount showed only negligible changes: +5 *q*_e_ and +4 *q*_e_, respectively. When the system was returned to vacuum, the charge reverted to a negative value of −3 *q*_e_, close to the previous negative charge state corresponding to Figure 2f. Thus, the combination of the structural analysis from the TEM images with the phase analysis from electron holography suggests that the structure change on the Au NP surface accompanying O_2_ gas injection and removal is strongly correlated with the charge state.

The phase changes induced by O_2_ gas injection were reproducible when other Au NPs on CeO_2_ were examined. Figure S4 (Supporting Information) demonstrated that three other Au NPs in vacuum had negative charges and that the negative charges of the Au NPs decreased in O_2_. Au NPs with single (Figure S4a–d, Supporting Information) and multiple domains (Figure S4e–h, Supporting Information) showed similar phase changes accompanying O_2_ gas injection and removal. A further small NP (Figure S4i–l, Supporting Information) showed only slight change with O_2_ injection, although the charge amount was certainly reduced. These results indicate that the introduction of O_2_ gas decreases the intrinsic negative charges of Au NPs on CeO_2_, although the amount of charge change could depend on the NP size.

**Figure 3:**
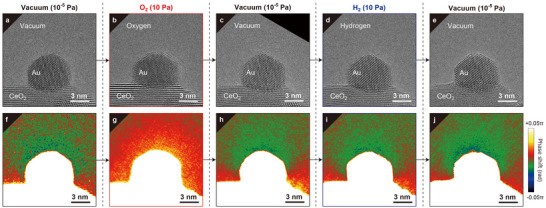
Structure and charge changes of Au/CeO_2_ in various gases. a–e) TEM images of Au/CeO_2_ in (a) vacuum, (b) 10 Pa O_2_, (c) vacuum after O_2_, (d) 10 Pa of H_2_, and (e) vacuum after H_2_. f–j) Noise‐reduced phase images corresponding to (a–e), respectively.

The effect of different types of gases on the surface reactions of the Au NP was investigated. For comparison with the observations for the representative oxidizing O_2_ gas shown in Figure 2, we introduced H_2_ gas as a reducing gas. **Figure** **3** presents the relationship between the atmosphere (i.e., vacuum, O_2_, and H_2_) and the structure/charge of the Au NP. As shown in Figure 3, the results were obtained under the following sequence of atmospheres: (a) vacuum, (b) O_2_, (c) vacuum, (d) H_2_, and (e) vacuum. The results for both O_2_ and H_2_ gases at 10 Pa are provided for comparison. Although the Au NP surface was disordered in the oxidizing environment of O_2_ (Figure 3b), it maintained an fcc‐ordered crystalline surface in the reducing environment of H_2_ (Figure 3d). A comparison of the phase images indicates that the negative charge of the supported Au NP decreased in O_2_ (from −4 *q*_e_ (Figure 3f) to +5 *q*_e_ (Figure 3g)), whereas the charge state in H_2_ was close to that in vacuum (−3 *q*_e_ in Figure 3h,i, −4 *q*_e_ in Figure 3j). The slight differences between the charges observed in vacuum (Figure 3f, h, and j) and H_2_ (Figure 3i) can be attributed to minor changes in the shape of the Au NP, which were induced during the collection of the data presented in Figure 3. These results reveal that both the surface structure and the charge state of a Au NP can be controlled via gas selection. Thus, we concluded that the structural changes on the Au NP surface during redox cycles were correlated with the change in the charge state of the NP.

**Figure 4:**
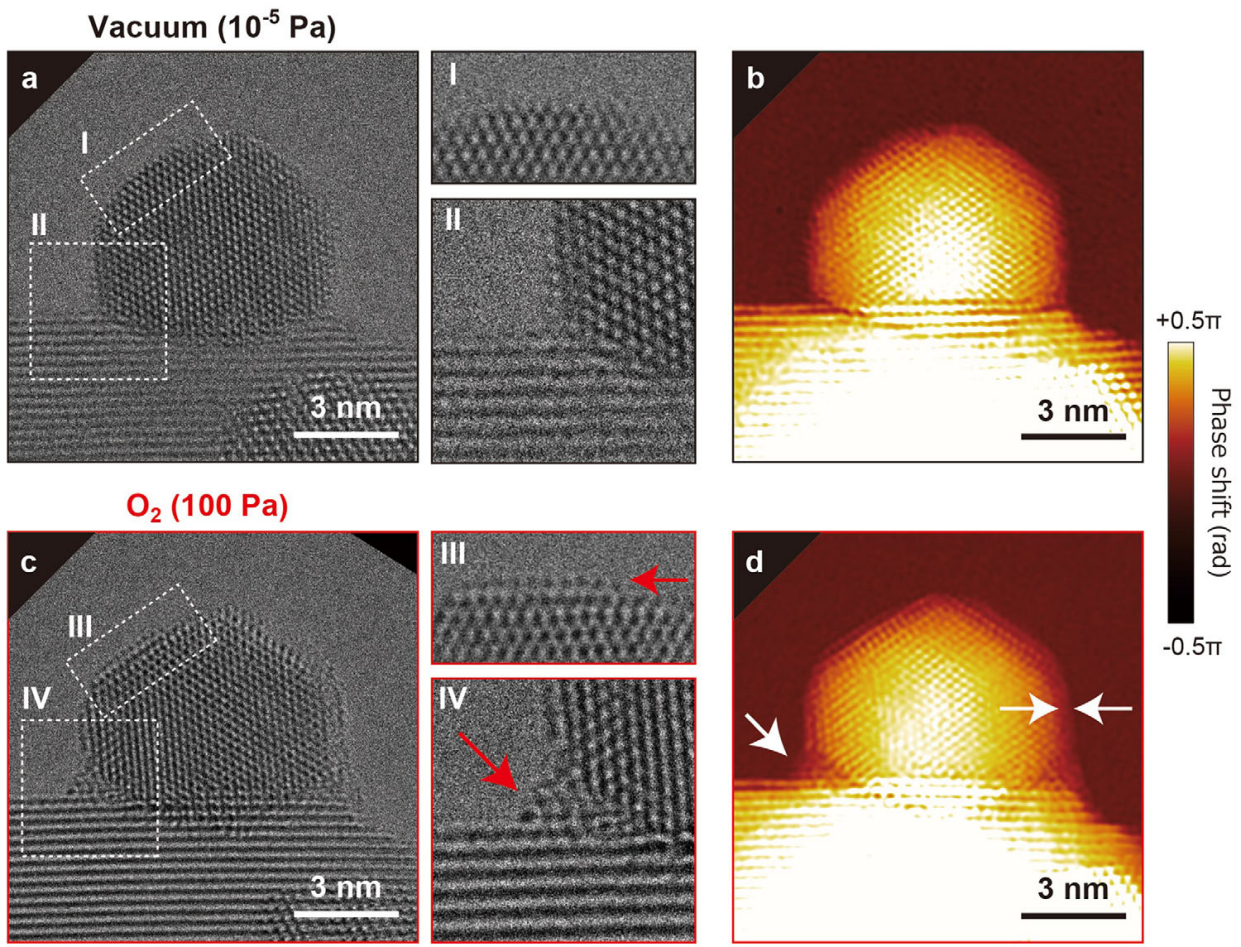
Structure and phase changes of Au NP surface and Au–CeO_2_ interface. a) TEM image of Au/CeO_2_ in vacuum. The surface and perimeter interface are represented in the rectangular regions indicated by I and II, respectively. The enlarged images of these regions are shown to the right of (a). c) TEM image of Au/CeO_2_ in 100 Pa of O_2_. The surface and perimeter interface are represented in the rectangular regions indicated by III and IV, respectively. The enlarged images of these regions are shown at the right of (c). The red arrows indicate the disordered regions. b,d) Noise‐reduced phase images corresponding to (a and c), respectively. The white arrows indicated the disordered regions.

The results in **Figure** **4**, the data for which were collected in vacuum and in 100 Pa of O_2_, enable a deeper understanding of the relationship between the changes in surface structures and changes in the charge states of the Au NP. In vacuum, the Au NP maintained an ordered crystal structure at the surface (Figure 4a). By contrast, when O_2_ gas was introduced, the surface structure became disordered, as indicated by the red arrow in region III in Figure 4c. This disordered surface reverted to an ordered surface in fcc‐Au crystal when the atmosphere was returned to vacuum. Thus, it is unlikely that the NP was coated by CeO_x_ formed via SMSI. SMSI is not readily attained with a CeO_2_ support,^[^ ^42^ ^]^ in contrast to the case of TiO_2_ support.^[^ ^44^ ^]^ Additionally, TiO_x_ formed through SMSI is robust under electron irradiation regardless of the gas atmosphere: that is, the TiOx layer remains intact even in the vacuum condition after removal of gases.^[^ ^44^ ^]^ Therefore, the surface disorder is primarily attributed to a structural change in the Au NP surface induced by oxygen species. A similar disordered region was produced at the perimeter of the metal–support interface, as indicated by the red arrow in region IV in Figure 4c. Previous studies^[^ ^42^, ^45^ ^]^ have suggested that the introduced O_2_ gas can cause a disturbance in the atomic arrangement at the perimeter interface. The phase image in Figure 4d, which was reconstructed at atomic resolution, provided valuable insights for understanding the structural disorder in the surface region of the Au NP, revealing a thickness of a few atomic layers. The disordered regions at the surface and the perimeter interface of the Au NP in O_2_ gas, indicated by the white arrows in Figure 4d, exhibited phase changes distinct from both the region outside of the NP and the fcc crystal structure inside of the NP. Considering these electron holography observations along with the previous reports,^[^ ^42^, ^45^ ^]^ it is likely that the disordered regions produced in O_2_ gas could be attributed to the reaction between Au and oxygen species, which formed a dynamic structure consisting of both Au and oxygen atoms such as a disordered gold oxide or an oxygen‐intercalated gold structure.^[^ ^54^, ^55^ ^]^ However, at the perimeter of the metal–support interface, the possibility of SMSI‐induced CeO_x_ contributing to the observed disorder cannot be ruled out, as SMSI may lead to the migration of oxide supports onto the NP surface near the interface under certain conditions.

### 2.3 Charge Analysis by DFT Calculations

**Figure 5:**
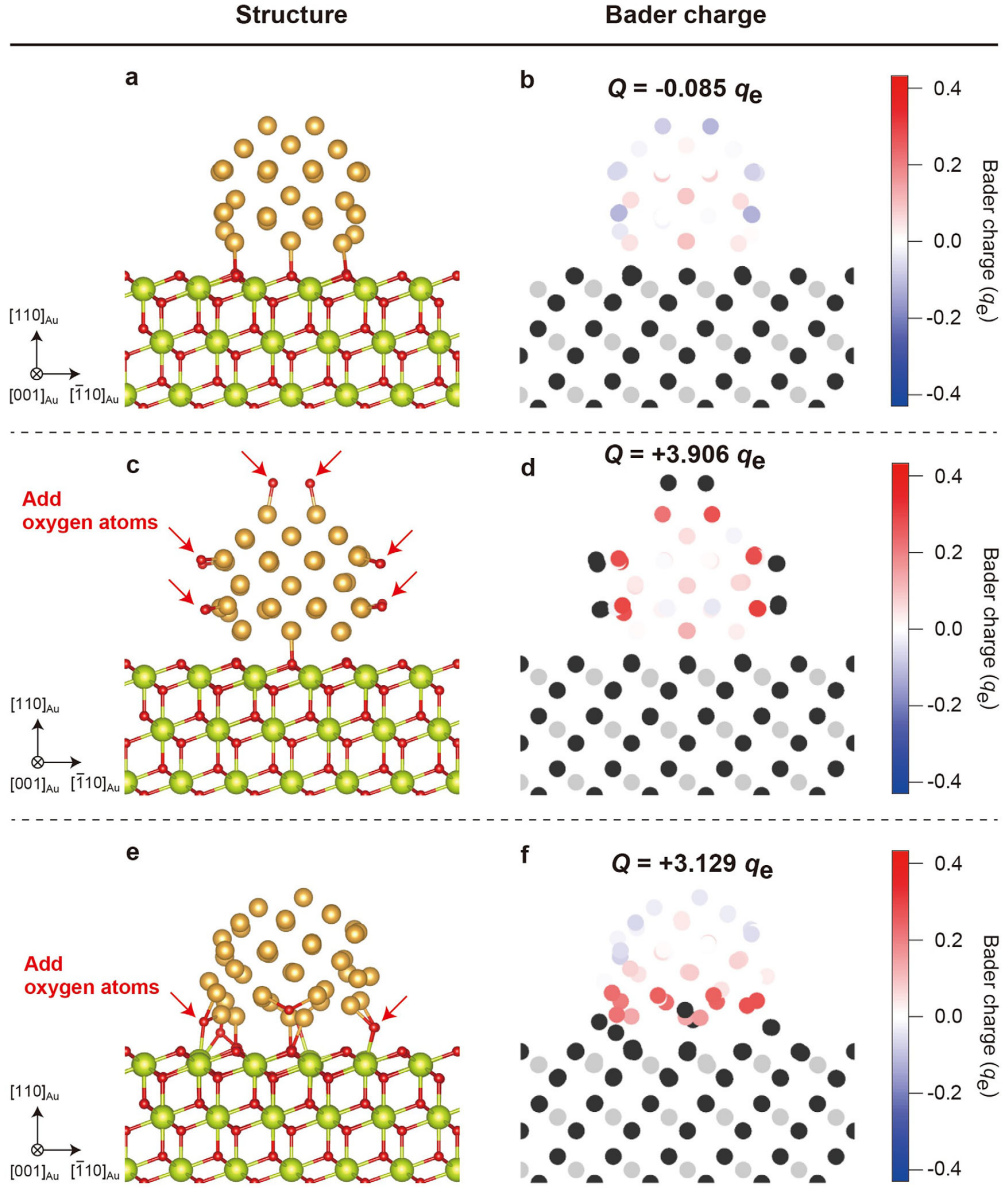
Charge analysis by DFT calculations. a,b) Structure (a) and Bader charge distribution (b) for a Au NP on a stoichiometric CeO_2_ (111) surface. c,d) Structure (c) and Bader charge distribution (d) for a Au NP, with oxygen atoms added on the Au NP surface. e,f) Structure (e) and Bader charge distribution (f) for a Au NP, with oxygen atoms added on the perimeter of the interface. The red and blue in the Bader charge distributions for the Au NP indicate the extent of charge accumulation (positive) and depletion (negative), respectively.

The changes in the charge states of the supported Au NPs in the presence of O_2_ gas were analyzed using density functional theory (DFT) calculations. An atomic model of a Au NP supported on the CeO_2_ (111) surface was constructed, and Bader charge analysis was performed (**Figure** **5**a,b). The charge of the Au NP (representing the NP free from the surface disordering in an oxygen environment) was *Q* = −0.085 *q*_e_, indicating a slightly negatively charged state, although the particle size assumed in the calculation (≈0.8 nm in diameter) was much smaller than that of the observation (≈6 nm in diameter). The charge transfer at the metal–support interface can be interpreted on the basis of the difference in work functions of the respective crystal planes at the interface. The work functions of bulk Au and CeO_2_ are 4.7 ≤ *φ*_Au_ ≤ 5.3 eV^[^ ^22^, ^26^, ^55^ ^]^ and *φ*_CeO2_ = 4.7 eV,^[^ ^56^ ^]^ respectively. Therefore, when the Au (110) plane is parallel to the CeO_2_ (111) plane, for the condition *φ*_CeO2_ < *φ*_Au_, electrons transfer from the CeO_2_ to the Au NP, explaining the negative charge of the Au NP.

To investigate the effect of oxygen adsorption, we constructed simple structure models of a Au NP with oxygen atoms added at the surface and at the perimeter of the metal–support interface. When oxygen atoms were adsorbed onto the surface (Figure 5c,d), the charge of the Au atoms adsorbed by oxygen atoms became positive, resulting in a positively charged Au NP. Similarly, when oxygen atoms were introduced at the perimeter interface (Figure 5e,f), the charge of the Au atoms near the interface also became positive, resulting in a positively charged Au NP. These results are consistent with the phase analysis results in Figure 2, where the negative charge of the Au NP shifted to a positive charge upon the introduction of O_2_ gas. The adsorption behavior of oxygen can be discussed on the basis of adsorption energy. Figure S5 (Supporting Information) shows the adsorption energies of oxygen at the surface top and perimeter interface of the Au NP supported on CeO_2_. The results indicate that the adsorption energy was lower at the perimeter interface, suggesting that the dissociative adsorption of oxygen molecules at the perimeter interface is preferentially promoted over adsorption at the surface in Au/CeO_2_, which has often been reported to be the location of active sites for catalytic reactions.^[^ ^11^, ^30^, ^41^, ^42^, ^57^ ^]^

### 2.4 Effects of Electron Irradiation and Contamination

The effects of electron irradiation and surface contamination on the electron holography observations should be discussed. Electron irradiation is well known to cause the emission of secondary electrons, which can make the specimen positively charged and/or induce radiation damage in the specimen. In the present study, to minimize the effects of electron irradiation, the current density of the electron beam was set relatively low at 4 A/cm^2^—a condition under which radiation‐induced damage, such as the SMSI in Au/TiO_2_ systems, can be negligible.^[^ ^44^ ^]^ As demonstrated with the Pt/TiO_2_ system,^[^ ^36^ ^]^ electron irradiation induces an artificial, positively charging in the metal NP due to the radiation damage to the oxide support crystal. In the Au/CeO_2_ system, similar phase changes induced by electron irradiation were demonstrated (Figure S6, Supporting Information). By estimating the phase shifts under electron exposure in various conditions, such as in vacuum, O_2_, and H_2_, we found that the phase shift slightly increased under all conditions, indicating positive charging. The phase‐shift increase concerning exposure time was 1.4 × 10^−5^, 8.9 × 10^−6^, and 2.0 × 10^−5^ rad s^−1^ in the vacuum, O_2_, and H_2_, respectively. The phase shift due to undesired radiation‐induced events during the collection of 100 holograms could be suppressed below 0.0014, 0.0009, and 0.0020 rad in the vacuum, O_2_, and H_2_, respectively. These results indicate that the radiation‐induced phase change is negligible compared with the phase shift induced by gas introduction targeted in this study. Thus, the observation of negative charging in vacuum reflects the intrinsic charge state of the Au NP. Additionally, the reversible changes in the crystal structure and charge state of the Au NP during gas injection and removal cycles suggest that the effect of electron irradiation on the Au/CeO_2_ system is negligibly small for this charge analysis. The introduced gas molecules can scatter the incident electron beam and adversely affect the image quality of electron holograms as well as TEM images. We confirmed that the image quality of electron holograms was maintained to 500 Pa for both O_2_ and H_2_ gas compared to that in vacuum, consistent with a previous report on electron holography in ETEM.^[^ ^48^ ^]^ Nevertheless, we used the data within the pressure range to 100 Pa for phase analysis because the sample drift during data collection was significant when the pressure exceeded 100 Pa.

The effect of contamination on the specimens was investigated. As shown in Figure S7a (Supporting Information), the surface of the Au NP before observation was coated with a ≈1 nm hydrocarbon‐based contamination layer. In ETEM observations of metallic nanomaterials, hydrocarbon‐based contamination can be removed by electron irradiation in an oxygen environment.^[^ ^55^ ^]^ As demonstrated in Figures S7c,d (Supporting Information), the phase change outside of the Au NP with surface contamination (S7c) was smaller than that without surface contamination (S7d). It appears that the conductive hydrocarbon‐based contamination layer enables charge diffusion to the support, resulting in the NP being closer to charge‐neutral. In the present study, because a clean Au surface without contamination was used for the gas experiments, the phase changes observed in the various gas environments reflect the changes in the intrinsic charge state on the NP surfaces accompanying gas reactions.

## 3 Conclusion

In summary, we achieved direct nanoscale visualization of the charge states of Au NPs supported on CeO_2_ during redox cycles using high‐precision electron holography in gas environments. The Au/CeO_2_ catalyst in vacuum exhibited a negative charge and an ordered and well‐defined fcc lattice over the whole region of Au NP. When oxidizing O_2_ gas was introduced, the surface region of the Au NP became disordered, accompanied by a decrease in negative charge (i.e., a shift toward positive charge). Conversely, when the reducing H_2_ gas was introduced, no substantial changes were observed in either the crystal structure or the charge state. By systematically varying the redox environments through gas injection and removal, we revealed that the surface crystal structure and charge state of the Au NP could be reversibly changed. The influence of oxygen was confirmed by DFT calculations, which demonstrated that both the Au NP surface and the perimeter of the metal–support interface could contribute to the practical catalytic reactions, such as CO oxidation. In particular, the surface disorder of Au NP plays a crucial role in controlling the charge state, thereby influencing the dissociative adsorption of oxygen molecules, which is a fundamental step in the reaction process. Our study demonstrates that the charge states of the Au NPs can be directly evaluated at the single‐particle level in gas environments using electron microscopy. This capability provides a unique opportunity to investigate the correlation between charge states and catalytic reactivity. In practical catalytic environments where multiple gases coexist, various chemical reactions occur simultaneously. Therefore, understanding surface reactions in single‐gas environments is essential as an initial step. To investigate how redox reactions at the surface influence charge states, we examined two representative redox gases—oxygen and hydrogen—separately. The differences in surface structures observed under oxygen and hydrogen atmospheres provide crucial insights into the stability of crystalline surface structures and the nature of active sites, particularly in relation to gas adsorption and desorption. These findings establish a fundamental basis for accurately interpreting subsequent CO oxidation reactions. To further advance this research, we expect that the direct and simultaneous elucidation of atomic‐level structures and charge states using a combination of electron holography and ETEM will pave the way for the characterization of practical nanomaterials under working environments such as real‐time, real‐space, and reactive gas environments in materials science and catalysis chemistry.

## Conflict of Interest

The authors declare no conflict of interest.

## Supporting information



Supporting Information

## Data Availability

The data that support the findings of this study are available from the corresponding author upon reasonable request.

## References

[1] J. Liu , L. Chen , X. Liu , ACS Catal. 2024, 14, 1987.

[2] L. Liu , A. Corma , Chem. Rev. 2018, 118, 4981. 29658707 10.1021/acs.chemrev.7b00776 PMC6061779

[3] M. S. Chen , D. W. Goodman , Science 2004, 306, 252. 15331772 10.1126/science.1102420

[4] N. Kamiuchi , K. Sun , R. Aso , M. Tane , T. Tamaoka , H. Yoshida , S. Takeda , Nat. Commun. 2018, 9, 2060. 29802253 10.1038/s41467-018-04412-4 PMC5970267

[5] B. Zugic , L. Wang , C. Heine , D. N. Zakharov , B. A. Lechner , E. A. Stach , J. Biener , M. Salmeron , R. J. Madix , C. M. Friend , Nat. Mater. 2016, 16, 558. 27992418 10.1038/nmat4824

[6] M. A. Sanchez‐Castillo , C. Couto , W. B. Kim , J. A. Dumesic , Angew. Chem., Int. Ed. 2004, 43, 1140. 10.1002/anie.200353238 14983457

[7] M. Haruta , T. Kobayashi , H. Sano , N. Yamada , Chem. Lett. 1987, 16, 405.

[8] M. Haruta , N. Yamada , T. Kobayashi , S. Iijima , J. Catal. 1989, 115, 301.

[9] M. Haruta , Catal. Today 1997, 36, 153.

[10] R. Si , M. Flytzani‐Stephanopoulos , Angew. Chem., Int. Ed. 2008, 47, 2884. 10.1002/anie.200705828 18327859

[11] M. Haruta , Faraday Discuss. 2011, 152, 11. 22455036 10.1039/c1fd00107h

[12] T. W. van Deelen , C. Hernández Mejía , K. P. de Jong , Nat. Catal. 2019, 2, 955.

[13] S. J. Tauster , S. C. Fung , R. L. Garten , J. Am. Chem. Soc. 1978, 100, 170.

[14] Z. Luo , G. Zhao , H. Pan , W. Sun , Adv. Energy Mater. 2022, 12, 2201395.

[15] C. T. Campbell , Nat. Chem. 2012, 4, 597. 22824888 10.1038/nchem.1412

[16] T. Binninger , T. J. Schmidt , D. Kramer , Phys. Rev. B 2017, 96, 165405.

[17] I. X. Green , W. J. Tang , M. Neurock , J. T. Yates , Science 2011, 333, 736. 21817048 10.1126/science.1207272

[18] Y. Lykhach , S. M. Kozlov , T. Skala , A. Tovt , V. Stetsovych , N. Tsud , F. Dvorak , V. Johanek , A. Neitzel , J. Myslivecek , S. Fabris , V. Matolin , K. M. Neyman , J. Libuda , Nat. Mater. 2016, 15, 284. 26657332 10.1038/nmat4500

[19] S. Porsgaard , P. Jiang , F. Borondics , S. Wendt , Z. Liu , H. Bluhm , F. Besenbacher , M. Salmeron , Angew. Chem., Int. Ed. 2011, 50, 2266. 10.1002/anie.201005377 21351333

[20] N. T. Khoa , S. W. Kim , D.‐H. Yoo , E. J. Kim , S. H. Hahn , Appl. Catal. A: Gen. 2014, 469, 159.

[21] N. Nilius , M. V. Ganduglia‐Pirovano , V. Brazdova , M. Kulawik , J. Sauer , H. J. Freund , Phys. Rev. Lett. 2008, 100, 096802. 18352741 10.1103/PhysRevLett.100.096802

[22] L. Olesen , M. Brandbyge , M. R. Sorensen , K. W. Jacobsen , E. Laegsgaard , I. Stensgaard , F. Besenbacher , Phys. Rev. Lett. 1996, 76, 1485. 10061735 10.1103/PhysRevLett.76.1485

[23] Q. Zhang , Y. J. Li , H. F. Wen , Y. Adachi , M. Miyazaki , Y. Sugawara , R. Xu , Z. H. Cheng , J. Brndiar , L. Kantorovich , I. Stich , J. Am. Chem. Soc. 2018, 140, 15668. 30403344 10.1021/jacs.8b07745

[24] T. Kittel , E. Roduner , J. Phys. Chem. C 2016, 120, 8907.

[25] Y. Zhang , O. Pluchery , L. Caillard , A. F. Lamic‐Humblot , S. Casale , Y. J. Chabal , M. Salmeron , Nano Lett. 2015, 15, 51. 25485557 10.1021/nl503782s

[26] N. Turetta , F. Sedona , A. Liscio , M. Sambi , P. Samorì , Adv. Mater. Interfaces 2021, 8, 2100068.

[27] T. Akita , M. Okumura , K. Tanaka , M. Kohyama , M. Haruta , Catal. Today 2006, 117, 62.

[28] H. Hojo , M. Nakashima , S. Yoshizaki , H. Einaga , ACS Nano 2024, 18, 4775. 38285709 10.1021/acsnano.3c09092

[29] R. Ishikawa , Y. Ueno , Y. Ikuhara , N. Shibata , Nano Lett. 2022, 22, 4161. 35533402 10.1021/acs.nanolett.2c00929

[30] M. Cargnello , V. V. T. Doan‐Nguyen , T. R. Gordon , R. E. Diaz , E. A. Stach , R. J. Gorte , P. Fornasiero , C. B. Murray , Science 2013, 341, 771. 23868919 10.1126/science.1240148

[31] S. Ichikawa , T. Akita , M. Okumura , M. Haruta , K. Tanaka , M. Kohyama , J. Electron Microsc. 2003, 52, 21. 10.1093/jmicro/52.1.21 12741484

[32] Y. Lu , F. Zheng , Q. Lan , M. Schnedler , P. Ebert , R. E. Dunin‐Borkowski , Nano. Lett. 2022, 22, 6936. 36041122 10.1021/acs.nanolett.2c01510

[33] F. Zheng , M. Beleggia , V. Migunov , G. Pozzi , R. E. Dunin‐Borkowski , Ultramicroscopy 2022, 241, 113593. 35944328 10.1016/j.ultramic.2022.113593

[34] C. Gatel , A. Lubk , G. Pozzi , E. Snoeck , M. Hytch , Phys. Rev. Lett. 2013, 111, 025501. 23889416 10.1103/PhysRevLett.111.025501

[35] T. Suzuki , S. Aizawa , T. Tanigaki , K. Ota , T. Matsuda , A. Tonomura , Ultramicroscopy 2012, 118, 21. 22728401 10.1016/j.ultramic.2012.04.007

[36] R. Aso , H. Hojo , Y. Takahashi , T. Akashi , Y. Midoh , F. Ichihashi , H. Nakajima , T. Tamaoka , K. Yubuta , H. Nakanishi , H. Einaga , T. Tanigaki , H. Shinada , Y. Murakami , Science 2022, 378, 202. 36227985 10.1126/science.abq5868

[37] C. Ophus , Microsc. Microanal. 2019, 25, 563. 31084643 10.1017/S1431927619000497

[38] M. J. Zachman , V. Fung , F. Polo‐Garzon , S. Cao , J. Moon , Z. Huang , D. E. Jiang , Z. Wu , M. Chi , Nat. Commun. 2022, 13, 3253. 35668115 10.1038/s41467-022-30923-2 PMC9170698

[39] Y. He , J. C. Liu , L. Luo , Y. G. Wang , J. Zhu , Y. Du , J. Li , S. X. Mao , C. Wang , Proc. Natl. Acad. Sci. USA 2018, 115, 7700. 29987052 10.1073/pnas.1800262115 PMC6065042

[40] N. Ta , J. J. Liu , S. Chenna , P. A. Crozier , Y. Li , A. Chen , W. Shen , J. Am. Chem. Soc. 2012, 134, 20585. 23267697 10.1021/ja310341j

[41] H. Yoshida , Y. Kuwauchi , J. R. Jinschek , K. J. Sun , S. Tanaka , M. Kohyama , S. Shimada , M. Haruta , S. Takeda , Science 2012, 335, 317. 22267808 10.1126/science.1213194

[42] T. Uchiyama , H. Yoshida , Y. Kuwauchi , S. Ichikawa , S. Shimada , M. Haruta , S. Takeda , Angew. Chem., Int. Ed. 2011, 50, 10157. 10.1002/anie.201102487 21910189

[43] S. T. Kuwauchi , H. Yoshida , K. Sun , M. Haruta , H. Kohno , Nano Lett. 2013, 13, 3073. 23786232 10.1021/nl400919c

[44] Y. Kuwauchi , H. Yoshida , T. Akita , M. Haruta , S. Takeda , Angew. Chem., Int. Ed. 2012, 51, 7729. 10.1002/anie.201201283 22730239

[45] S. Takeda , Y. Kuwauchi , H. Yoshida , Ultramicroscopy 2015, 151, 178. 25498142 10.1016/j.ultramic.2014.11.017

[46] T. W. Hansen , J. B. Wagner , Microsc. Microanal. 2012, 18, 684. 22691205 10.1017/S1431927612000293

[47] H. G. Liao , D. Zherebetskyy , H. L. Xin , C. Czarnik , P. Ercius , H. Elmlund , M. Pan , L. W. Wang , H. M. Zheng , Science 2014, 345, 916. 25146287 10.1126/science.1253149

[48] J. A. E. Hyllested , M. Beleggia , Ultramicroscopy 2021, 221, 113178. 33302046 10.1016/j.ultramic.2020.113178

[49] M. Schreiber , C. Cassidy , Microsc. Microanal. 2023, 29, 1575.

[50] P. Haluai , M. R. McCartney , P. A. Crozier , Microsc. Microanal. 2022, 28, 1906.

[51] S. Shimada , T. Takei , T. Akita , S. Takeda , M. Haruta , Stud. Surf. Sci. Catal. 2010, 175, 843.

[52] H. Lichte , M. Lehmann , Rep. Prog. Phys. 2008, 71, 016102.

[53] Y. Midoh , K. Nakamae , Microscopy 2020, 69, 123. 31977048 10.1093/jmicro/dfz115

[54] T. Bar , T. V. de Bocarme , B. E. Nieuwenhuys , N. Kruse , Catal. Lett. 2001, 74, 127.

[55] R. Aso , Y. Ogawa , T. Tamaoka , H. Yoshida , S. Takeda , Angew. Chem., Int. Ed. 2019, 58, 16028. 10.1002/anie.201907679 31486177

[56] A. Pfau , K. D. Schierbaum , Surf. Sci. 1994, 321, 71.

[57] D. Widmann , R. J. Behm , Acc. Chem. Res. 2014, 47, 740. 24555537 10.1021/ar400203e

