# Supplementary material for: Direct Visualization of Surface Structure and Charge States of Ceria‐Supported Gold Catalysts Under Redox Conditions
Source: Adv Sci (Weinh). 2025 Jul 10;12(36):e08554. doi: 10.1002/advs.202508554 (PMC12463076; doi:10.1002/advs.202508554)
Supplement: Supplementary file 1 — Supporting Information [file ADVS-12-e08554-s001.pdf]

ADVANCED SCIENCE
Open Access

Supporting Information

for *Adv. Sci.*, DOI 10.1002/advs.202508554

Direct Visualization of Surface Structure and Charge States of Ceria-Supported Gold Catalysts Under Redox Conditions

Ryotaro Aso, Takehiro Tamaoka, Hideto Yoshida, Hajime Hojo, Hiroki Sano, Yoshihiro Midoh, Hisahiro Einaga, Toshiaki Tanigaki and Yasukazu Murakami**

Supporting Information for

Direct Visualization of Surface Structure and Charge States of Ceria-Supported Gold Catalysts Under Redox Conditions

Ryotaro Aso,*[a] Takehiro Tamaoka,†[b] Hideto Yoshida,[c] Hajime Hojo,[d] Hiroki Sano,[a] Yoshihiro Midoh,[e] Hisahiro Einaga,[d] Toshiaki Tanigaki,[f] and Yasukazu Murakami*[a,b]

[a]Department of Applied Quantum Physics and Nuclear Engineering, Kyushu University, 744 Motooka, Nishi-ku, Fukuoka 819-0395, Japan.

[b]The Ultramicroscopy Research Center, Kyushu University, 744 Motooka, Nishi-ku, Fukuoka 819-0395, Japan.

[c]SANKEN, The University of Osaka, 8-1 Mihogaoka, Ibaraki, Osaka, 567-0047, Japan.

[d]Department of Advanced Materials Science and Engineering, Faculty of Engineering Sciences, Kyushu University, 6-1 Kasuga-koen, Kasuga, Fukuoka 816-8580, Japan.

[e]Graduate School of Information Science and Technology, The University of Osaka, 1-5 Yamadaoka, Suita, Osaka 565-0871, Japan.

[f]Research and Development Group, Hitachi, Ltd., 2520 Hatoyama, Saitama 350-0395, Japan.

†Present address: Morphological Research Laboratory, Toray Research Center, Inc., 3-2-11, Sonoyama, Otsu, Shiga 520-8567, Japan.

*Corresponding authors. Emails:

aso.ryotaro.072@m.kyushu-u.ac.jp (R.A.),

murakami.yasukazu.227@m.kyushu-u.ac.jp (Y.Murakami)

The Supporting Information includes:

Materials and Methods

Figures S1–S7

References

Materials and Methods

S1. Sample preparation

The Au nanoparticles (NPs) on CeO_2 were prepared using the following deposition–precipitation method.[51] An aqueous solution of $HAuCl_4$ (1×10^{-3} mol/L) was adjusted to pH 7 by adding NaOH, and the resultant solution was heated to 70°C. CeO_2 powder was then added, and the suspension was stirred at pH 7 and 70°C for 1 h. The suspension was centrifuged and washed to remove Cl and Na ions. The sample was collected by vacuum filtration and dried at 120°C overnight before being calcined in air at 300°C for 4 h.

S2. ETEM observations

The samples were observed using a spherical aberration-corrected environmental transmission electron microscopy (ETEM) apparatus (Thermo Fisher Scientific Titan ETEM G2; accelerating voltage: 300 kV) equipped with a specially designed environmental cell.[45] Although the partial pressures of the O_2 and H_2 gases were varied to pressures as high as 500 Pa, the data were obtained within the pressure range of 10^{-5}–100 Pa because of sample drift. The electron current density at the specimen position for ETEM imaging was set at 4 A/cm^2 to suppress undesired effects of electron irradiation, such as the strong metal–support interaction (SMSI), during observations of NP catalysts.[44] TEM images and electron holograms were acquired using an electron direct-detection K3 camera (Gatan). All measurements and observations were performed at room temperature (22°C).

The structure of Au/CeO_2 was characterized by TEM observations. The Au NPs with particle sizes smaller than ~10 nm were dispersed on CeO_2 supports (Figure S1). In this study, we selected a single Au NP supported at the edge of the flat CeO_2 (111) surface (Figure 1).

S3. Electron holography

Electron holography in gas environments was conducted using the ETEM apparatus equipped with a spherical aberration corrector, a biprism interferometer, and an electron direct-detection camera. The electron current density at the specimen position for electron holography was set at 4 A/cm^2. Electron holograms were collected from a region of 33.8 × 33.8 nm^2 (3456 × 3456 pixels) that contained a Au NP and part of the CeO_2 support. The typical interference fringe pitch was 0.11 nm (Figure 1d).

The image distortion in electron holograms at the sample was reduced by subtracting the phase image collected in the field of view (i.e., reference phase image). Additionally, we devised the following hologram collection scheme to avoid undesired radiation-induced events, such as the effects of secondary electron emission

and radiation damage. First, 25 holograms of the sample were continuously collected twice, each with an exposure time of 1 s. The sample was then removed from the field of view, and 25 reference holograms were collected twice, each with an exposure time of 1 s. We repeated this scheme twice under each measurement condition, such as in vacuum and under various gases.

To suppress the undesired radiation-induced events in the phase analysis, such as positive charging through electron emission shown in Figure S6, the stable-collected set of 25 holograms was used to evaluate the distributions of the electric field and charges in the phase images. We corrected the 25 holograms of the sample for image distortion by subtracting the reference holograms and transforming the corrected holograms into complex form. The noise reduction method using a wavelet hidden Markov model (WHMM)[36,53] was applied to the complex form, and the noise-reduced complex data were reconstructed into phase images. After being corrected for the relative drifts, the reconstructed and noise-reduced phase images were averaged (superimposed). Notably, the reproducibility experiments for other Au NPs (Figure S4) were performed using phase images with denoising applied to single-shot holograms (without image averaging) due to time-consuming data collection.

S4. Charge estimation of supported Au NPs

To estimate the charges on a single Au NP, we assumed that a point charge, Q, existed at the center of the spherical Au NP. We extrapolated three lines crossing at the central point of the Au NP and estimated the charges using curve fitting based on the charged sphere model,[58] as shown in Figure S2 (in vacuum) and Figure S3 (in gases). The average total charges of the Au NP, Q_{ave}, were calculated by averaging the results from the three lines. The root-mean-squared error (RMSE) was used as a measure of the precision in the phase analysis.

S5. DFT calculations

Spin-polarized density functional theory (DFT) calculations were conducted using the projector augmented wave potentials, as implemented in the Vienna Ab initio Simulation Package (VASP) code.[59,60] The generalized gradient approximation with the PBEsol parameterization was used to describe the exchange-correlation interaction.[61] To describe the strong on-site Coulomb repulsion of the Ce 4f electrons, we added the Durarev +U term with an effective value of 5.0 eV.[28,62] The cutoff energy for the plane-wave basis was 400 eV. After optimizing the structure of the CeO_2 unit cell, we constructed a slab model of the (111) surface with an in-plane size and thickness of ~22.9 Å × ~19.8 Å × ~7.8 Å. The vacuum space above the slab was 25 Å thick. According to the TEM observation results, Au atoms were arranged on the CeO_2 (111) surface such that the Au (110) plane was parallel to the CeO_2 (111) plane,

resulting in the formation of a symmetric NP composed of 44 Au atoms before relaxation. The in-plane orientation relationship was Au[110]//CeO_2[1-10]. All atoms in the cell were relaxed except for those in the bottom two atomic layers of CeO_2, which were fixed in bulk positions. The electronic energies were converged to 10^{-4} eV, and the ionic relaxations were considered to converge when the forces on the ions were less than 0.02 eV/Å. *k*-point sampling was performed at the Γ-point only. Dipole correction of the surface models was included in the surface normal direction. Ten oxygen atoms were placed on the surface of a Au NP in the case of the model structure shown in Figure 5c, and six oxygen atoms were placed at the perimeter of the Au–CeO_2 interface in the case of the model structure shown in Figure 5e. The atomic charges were estimated using Bader's analysis[63] on the basis of the numerical implementation developed by Henkelman *et al.*[64] As shown in Figure S5, the adsorption energies of an oxygen atom (E_{ads}) were calculated using the equation $E_{ads} = E(Au/CeO_2{+}O) - E(Au/CeO_2) - 1/2\ E(O_2)$, where $E(Au/CeO_2{+}O)$, $E(Au/CeO_2)$, and $E(O_2)$ are the calculated energies of the Au/CeO_2 slab model with an oxygen atom on it, the bare Au/CeO_2, and an oxygen molecule, respectively.

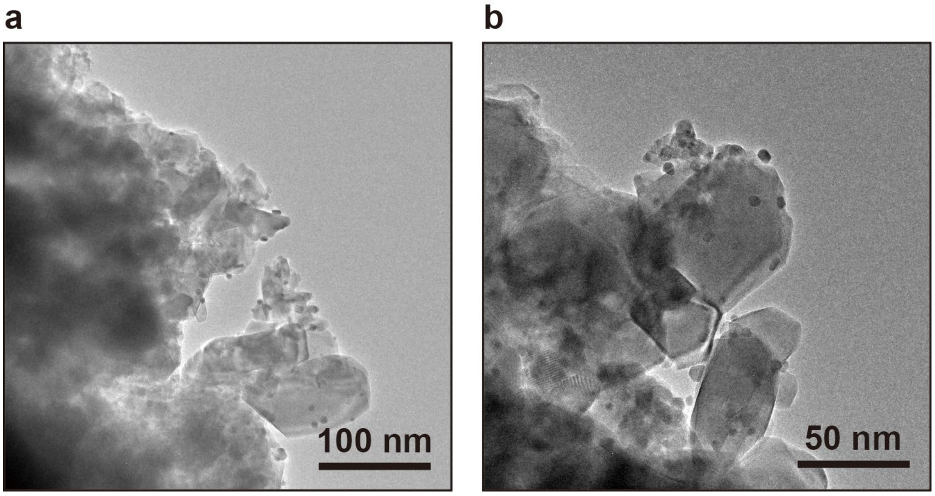

Figure S1. Structures of Au/CeO_2 catalysts. a,b) TEM images taken at (a) low and (b) high magnification.

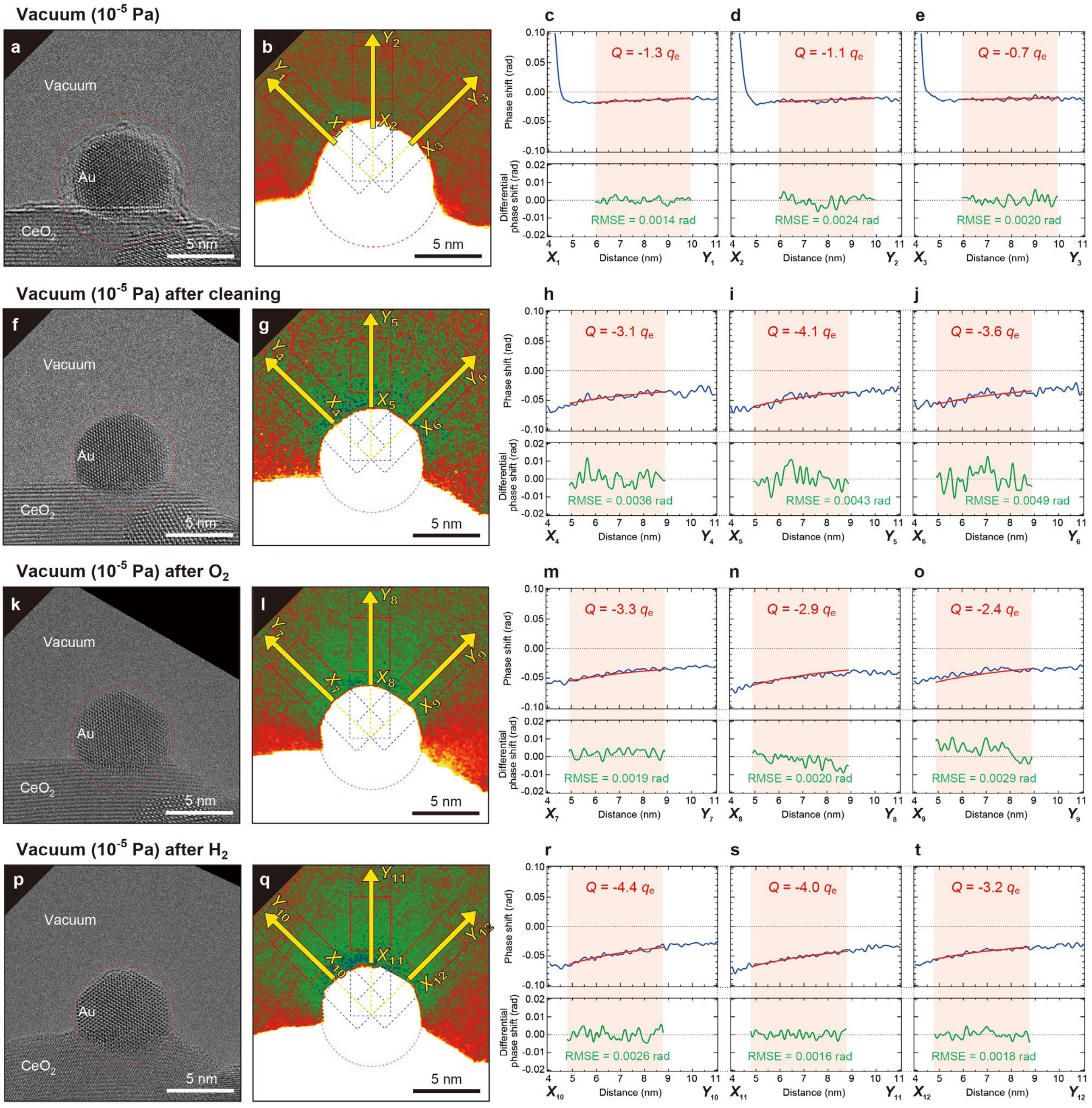

Figure S2. Quantification of charges on various Au NPs in vacuum. a,f,k,p) TEM images of Au/CeO_2 in (a) vacuum, (f) vacuum after cleaning, (k) vacuum after O_2, and (p) vacuum after H_2. b,g,l,q) Noise-reduced phase images obtained from specimens shown in (a, f, k, and p), respectively. c)–e) Plots of phase shifts along lines X_1–Y_1 (c), X_2–Y_2 (d), and X_3–Y_3 (e) in (b). h–j) Plots of phase shifts along lines X_4–Y_4 (h), X_5–Y_5 (i), and X_6–Y_6 (j) in (g). m–o) Plots of phase shifts along lines X_7–Y_7 (m), X_8–Y_8 (n), and X_9–Y_9 (o) in (l). r–t) Plots of phase shifts along lines X_{10}–Y_{10} (r), X_{11}–Y_{11} (s), and X_{12}–Y_{12} (t) in (q). The upper panels provide the phase plots, where the red lines show the results of curve fitting (applied to the hatched region) to evaluate the net charges Q of Au NPs. The lower panels provide the differences between the observations and the curve fitting results. The root-mean-squared error (RMSE) was used as a measure of the precision in the phase analysis.

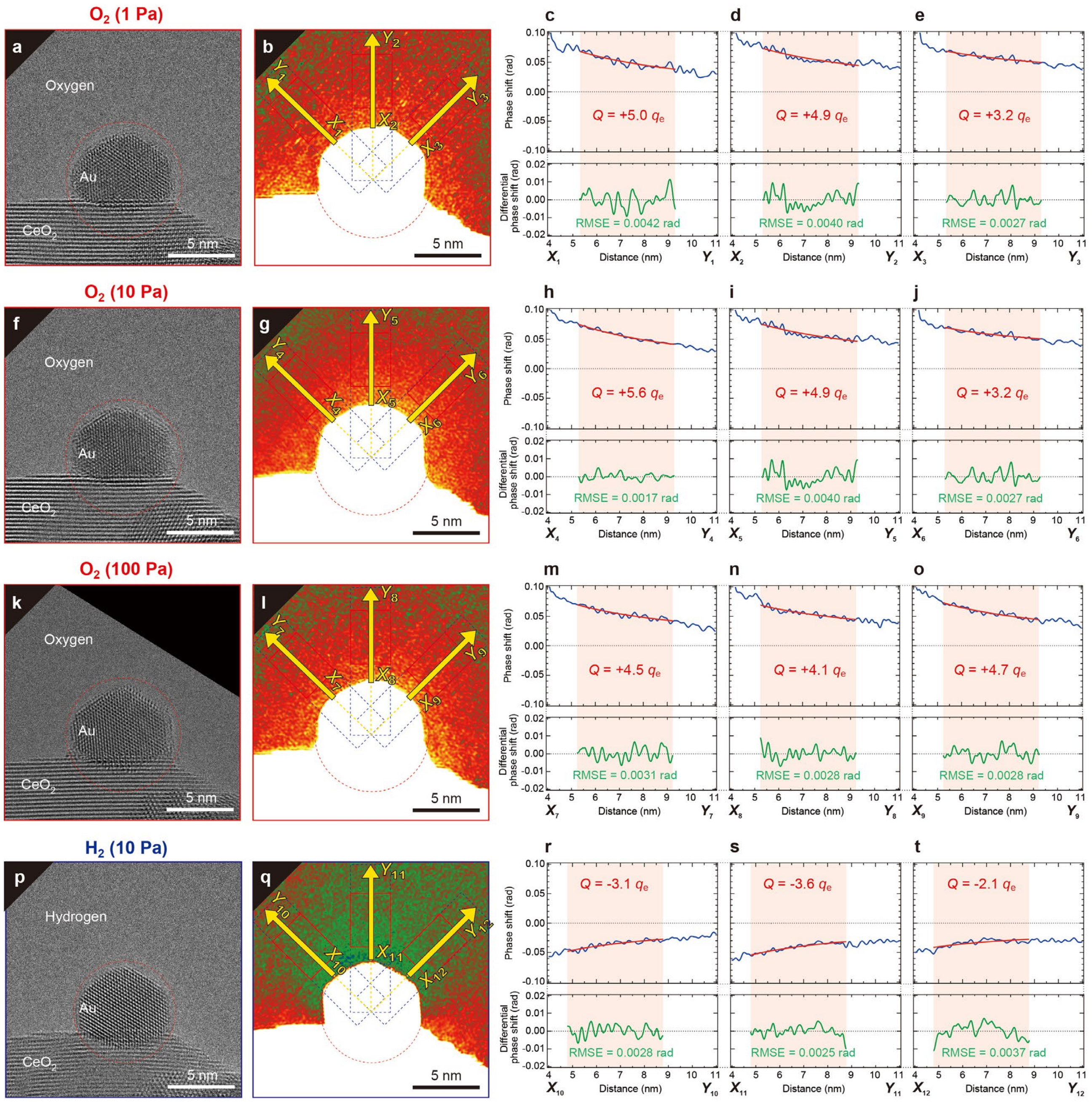

Figure S3. Quantification of charges on various Au NPs in gas. a,f,k,p) TEM images of Au/CeO_2 in (a) 1 Pa, (f) 10 Pa, and (k) 100 Pa of O_2 and in (p) 10 Pa of H_2. b,g,l,q) Noise-reduced phase images obtained from specimens shown in (a, f, k, and p), respectively. c–e) Plots of phase shifts along lines X_1–Y_1 (c), X_2–Y_2 (d), and X_3–Y_3 (e) in (b). h–j) Plots of phase shifts along lines X_4–Y_4 (h), X_5–Y_5 (i), and X_6–Y_6 (j) in (g). m–o) Plots of phase shifts along lines X_7–Y_7 (m), X_8–Y_8 (n), and X_9–Y_9 (o) in (l). r–t) Plots of phase shifts along lines X_{10}–Y_{10} (r), X_{11}–Y_{11} (s), and X_{12}–Y_{12} (t) in (q). The upper panels provide the phase plots, where the red lines show the results of curve fitting (applied to the hatched region) to evaluate the net charges Q of the Au NPs. The lower panels provide the differences between the observations and the curve fitting results. The RMSE was used as a measure of the precision in the phase analysis.

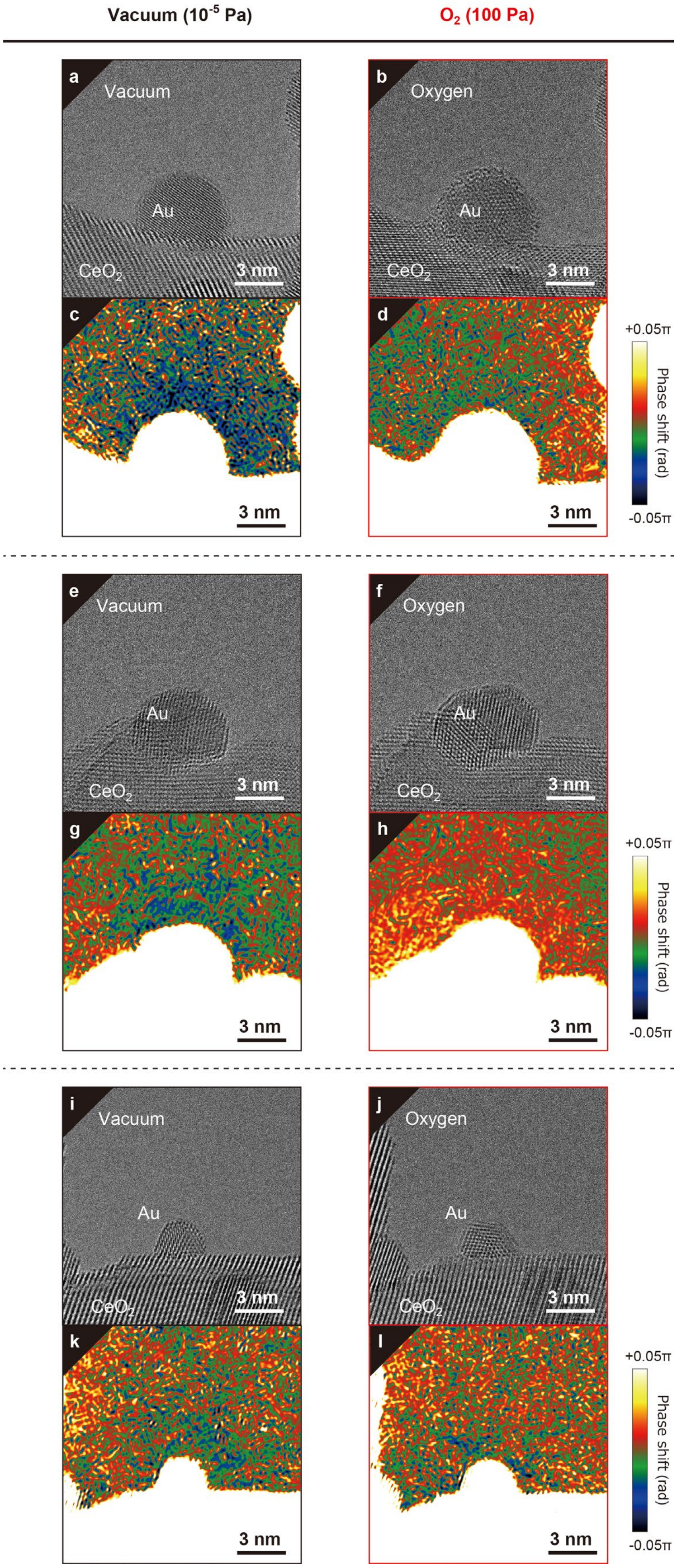

Figure S4. Structure and charge changes of various Au NPs on CeO_2 in O_2 gas. a,b,e,f,i,j) TEM images of three Au NPs on CeO_2 in (a, e, and i) vacuum and (b, f, and j) 100 Pa of O_2, respectively. c,d,g,h,k,l) Noise-reduced phase images corresponding to (a, b, e, f, i, and j), respectively.

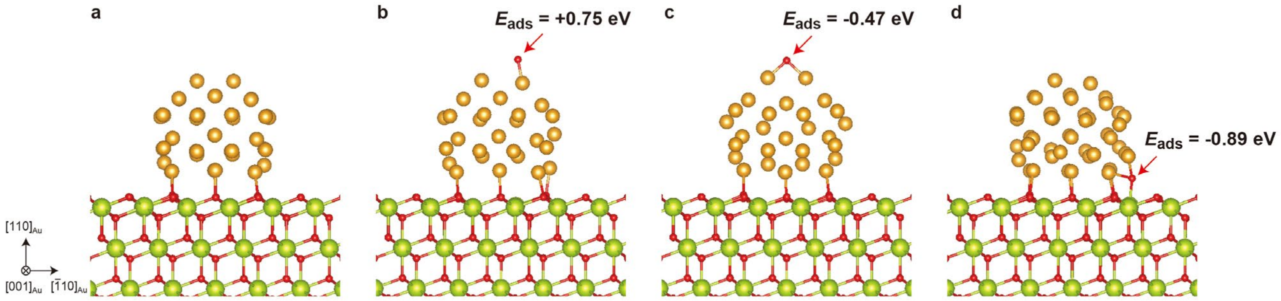

Figure S5. Adsorption energy of oxygen on Au NPs. a) Structure of Au NP on a stoichiometric CeO_2 (111) surface. b) Structure of a Au NP with an oxygen atom added on the top surface, where the Au NP is on a stoichiometric CeO_2 (111) surface. c) Structure of a Au NP with an oxygen atom bridging two Au atoms on the top surface, where the Au NP is on a stoichiometric CeO_2 (111) surface. d) Structure of a Au NP with an oxygen atom bridging the perimeter interface, where the Au NP is on a stoichiometric CeO_2 (111) surface. The adsorption energy of the adsorbed oxygen atom is indicated in (b–d).

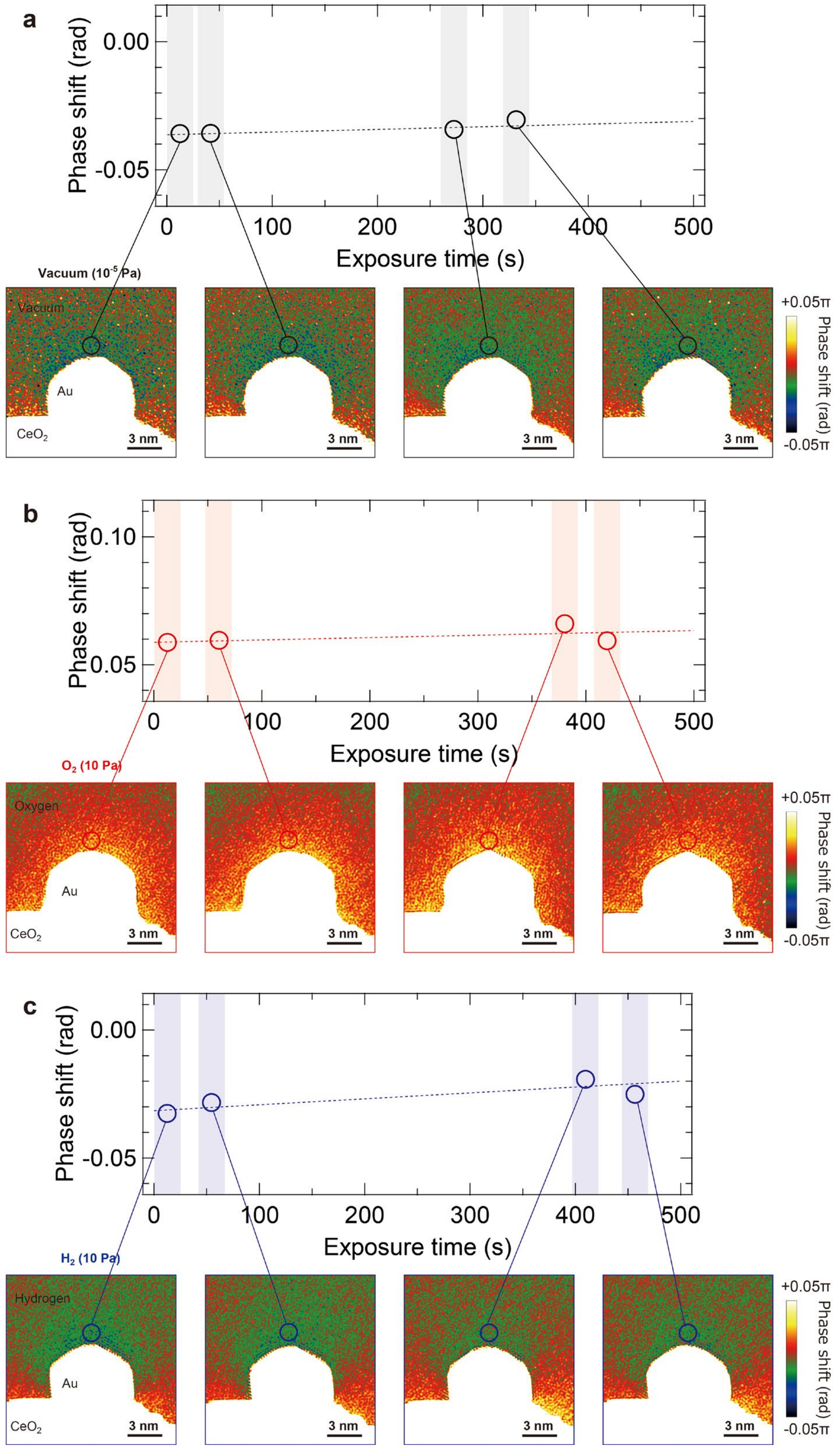

Figure S6. Examination of the relationship between phase shift and electron beam exposure time. a–c) Phase shifts observed at the top of a Au NP in (a) vacuum, (b) 10 Pa of O_2, and (c) 10 Pa of H_2 as a function of the exposure time. Each of the plots represents the average from 25 phase images in the black, red, and blue regions. The averaged phase images of the Au NP observed in the plots are inset. Each of the dashed lines is the linear approximation line.

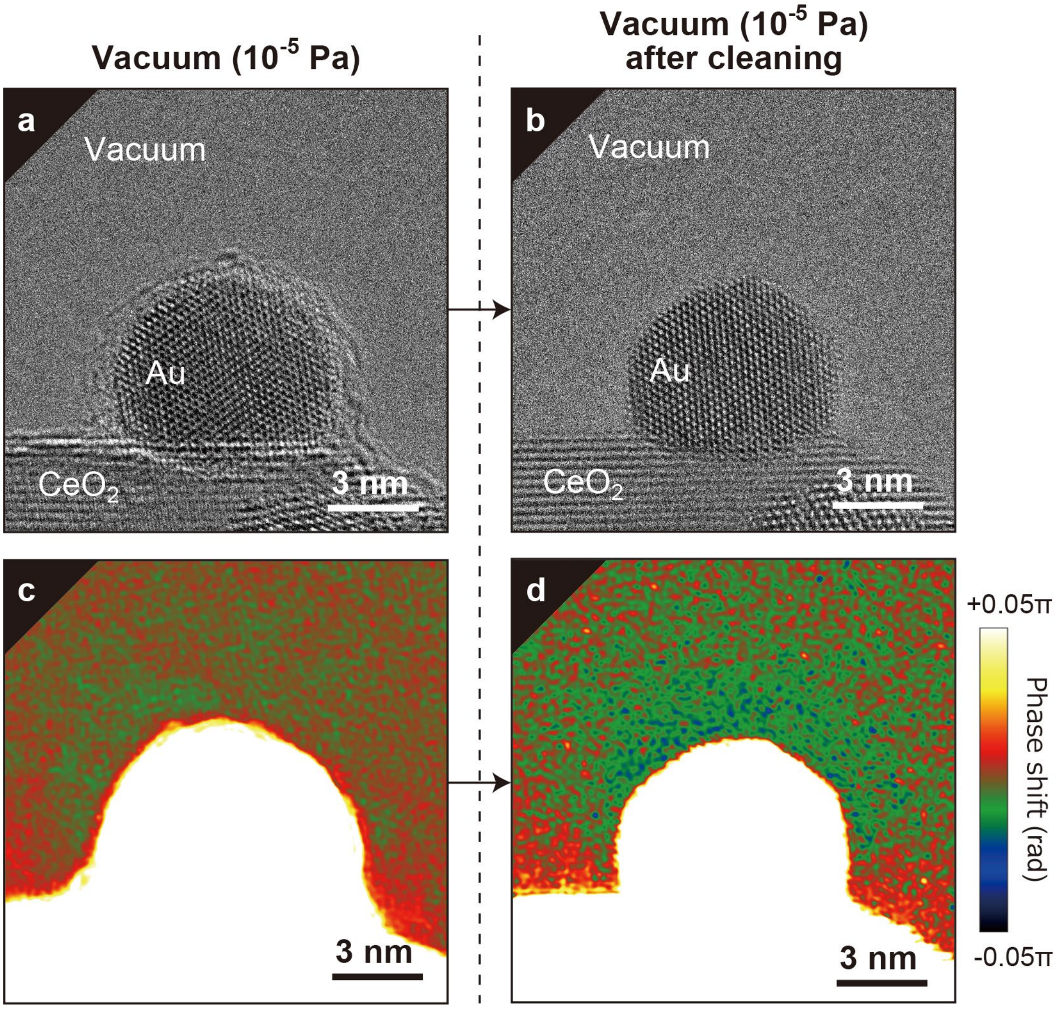

Figure S7. Structure and charge changes of Au/CeO_2 in vacuum. a) TEM image of Au/CeO_2 in vacuum before cleaning (with contamination). b) TEM image of Au/CeO_2 in vacuum after cleaning (without contamination). c,d) Noise-reduced phase images corresponding to (a and b), respectively.

References

[1] J. Liu, L. Chen, X. Liu, "Deep insight into characterizing the metal–support interface in heterogeneous catalysis." *ACS Catalysis* **2024**, *14*, 1987–2002.

[2] L. Liu, A. Corma, "Metal catalysts for heterogeneous catalysis: From single atoms to nanoclusters and nanoparticles." *Chem. Rev.* **2018**, *118*, 4981–5079.

[3] M. S. Chen, D. W. Goodman, "The structure of catalytically active gold on titania." *Science* **2004**, *306*, 252–255.

[4] N. Kamiuchi, K. Sun, R. Aso, M. Tane, T. Tamaoka, H. Yoshida, S. Takeda, "Self-activated surface dynamics in gold catalysts under reaction environments." *Nat. Commun.* **2018**, *9*, 2060.

[5] B. Zugic, L. Wang, C. Heine, D. N. Zakharov, B. A. Lechner, E. A. Stach, J. Biener, M. Salmeron, R. J. Madix, C. M. Friend, "Dynamic restructuring drives catalytic activity on nanoporous gold-silver alloy catalysts." *Nat. Mater.* **2016**, *16*, 558–564.

[6] M. A. Sanchez-Castillo, C. Couto, W. B. Kim, J. A. Dumesic, "Gold-nanotube membranes for the oxidation of CO at gas-water interfaces." *Angew. Chem. Int. Ed.* **2004**, *43*, 1140–1142.

[7] M. Haruta, T. Kobayashi, H. Sano, N. Yamada, "Novel gold catalysts for the oxidation of carbon-monoxide at a temperature far below 0°C." *Chem. Lett.* **1987**, *16*, 405–408.

[8] M. Haruta, N. Yamada, T. Kobayashi, S. Iijima, "Gold catalyst prepared by coprecipitation for low-temperature oxidation of hydrogen and of carbon-nomoxide." *J. Catal.* **1989**, *115*, 301–309.

[9] M. Haruta, "Size- and support-dependency in the catalysis of gold." *Catal. Today* **1997**, *36*, 153–166.

[10] R. Si, M. Flytzani-Stephanopoulos, "Shape and crystal-plane effects of nanoscale ceria on the activity of Au-CeO_2 catalysts for the water-gas shift reaction." *Angew. Chem. Int. Ed.* **2008**, *47*, 2884–2887.

[11] M. Haruta, "Spiers Memorial Lecture. Role of perimeter interfaces in catalysis by gold nanoparticles." *Faraday Discuss* **2011**, *152*, 11–32.

[12] T. W. van Deelen, C. Hernández Mejía, K. P. de Jong, "Control of metal-support interactions in heterogeneous catalysts to enhance activity and selectivity." *Nat. Catal.* **2019**, *2*, 955–970.

[13] S. J. Tauster, S. C. Fung, R. L. Garten, "Strong metal-support interactions--Group 8 novel metals supported on TiO_2." *J. Am. Chem. Soc.* **1978**, *100*, 170–175.

[14] Z. Luo, G. Zhao, H. Pan, W. Sun, "Strong metal–support interaction in heterogeneous catalysts." *Adv. Energy Mater.* **2022**, *12*, 2201395.

[15] C. T. Campbell, "Catalyst-support interactions: Electronic perturbations." *Nat. Chem.* **2012**, *4*, 597–598.

[16] T. Binninger, T. J. Schmidt, D. Kramer, "Capacitive electronic metal-support interactions: Outer surface charging of supported catalyst particles." *Phys. Rev. B* **2017**, *96*, 165405.

[17] I. X. Green, W. J. Tang, M. Neurock, J. T. Yates, "Spectroscopic observation of dual catalytic sites during oxidation of CO on a Au/TiO_2 Catalyst." *Science* **2011**, *333*, 736–739.

[18] Y. Lykhach, S. M. Kozlov, T. Skala, A. Tovt, V. Stetsovych, N. Tsud, F. Dvorak, V. Johanek, A. Neitzel, J. Myslivecek, S. Fabris, V. Matolin, K. M. Neyman, J. Libuda, "Counting electrons on supported nanoparticles." *Nat. Mater.* **2016**, *15*, 284–288.

[19] S. Porsgaard, P. Jiang, F. Borondics, S. Wendt, Z. Liu, H. Bluhm, F. Besenbacher, M. Salmeron, "Charge state of gold nanoparticles supported on titania under oxygen pressure." *Angew. Chem. Int. Ed.* **2011**, *50*, 2266–

2269.

[20] N. T. Khoa, S. W. Kim, D.-H. Yoo, E. J. Kim, S. H. Hahn, "Size-dependent work function and catalytic performance of gold nanoparticles decorated graphene oxide sheets." *Appl. Catal. A: Gen.* **2014**, *469*, 159–164.

[21] N. Nilius, M. V. Ganduglia-Pirovano, V. Brazdova, M. Kulawik, J. Sauer, H. J. Freund, "Counting electrons transferred through a thin alumina film into Au chains." *Phys. Rev. Lett.* **2008**, *100*, 096802.

[22] L. Olesen, M. Brandbyge, M. R. Sorensen, K. W. Jacobsen, E. Laegsgaard, I. Stensgaard, F. Besenbacher, "Apparent barrier height in scanning tunneling microscopy revisited." *Phys. Rev. Lett.* **1996**, *76*, 1485–1488.

[23] Q. Zhang, Y. J. Li, H. F. Wen, Y. Adachi, M. Miyazaki, Y. Sugawara, R. Xu, Z. H. Cheng, J. Brndiar, L. Kantorovich, I. Stich, "Measurement and manipulation of the charge state of an adsorbed oxygen adatom on the rutile TiO_2(110)-1×1 Surface by nc-AFM and KPFM." *J. Am. Chem. Soc.* **2018**, *140*, 15668–15674.

[24] T. Kittel, E. Roduner, "Charge polarization at catalytic metal–support junctions, Part A: Kelvin probe force microscopy results of noble metal nanoparticles." *J. Phys. Chem. C* **2016**, *120*, 8907–8916.

[25] Y. Zhang, O. Pluchery, L. Caillard, A. F. Lamic-Humblot, S. Casale, Y. J. Chabal, M. Salmeron, "Sensing the charge state of single gold nanoparticles via work function measurements." *Nano Lett.* **2015**, *15*, 51–55.

[26] N. Turetta, F. Sedona, A. Liscio, M. Sambi, P. Samorì, "Au(111) surface contamination in ambient conditions: Unravelling the dynamics of the work function in air." *Adv. Mater. Interfaces* **2021**, *8*, 2100068.

[27] T. Akita, M. Okumura, K. Tanaka, M. Kohyama, M. Haruta, "Analytical TEM observation of Au nano-particles on cerium oxide." *Catal. Today* **2006**, *117*, 62–68.

[28] H. Hojo, M. Nakashima, S. Yoshizaki, H. Einaga, "Lattice-plane-dependent distribution of Ce^{3+} at Pt and CeO_2 interfaces for Pt/CeO_2 catalysts." *ACS Nano* **2024**, *18*, 4775–4782.

[29] R. Ishikawa, Y. Ueno, Y. Ikuhara, N. Shibata, "Direct observation of atomistic reaction process between Pt nanoparticles and TiO_2 (110)." *Nano Lett.* **2022**, *22*, 4161–4167.

[30] M. Cargnello, V. V. T. Doan-Nguyen, T. R. Gordon, R. E. Diaz, E. A. Stach, R. J. Gorte, P. Fornasiero, C. B. Murray, "Control of metal nanocrystal size reveals metal-support interface role for ceria catalysts." *Science* **2013**, *341*, 771–773.

[31] S. Ichikawa, T. Akita, M. Okumura, M. Haruta, K. Tanaka, M. Kohyama, "Electron holographic 3-D nano-analysis of Au/TiO_2 catalyst at interface." *J. Electron Microsc.* **2003**, *52*, 21–26.

[32] Y. Lu, F. Zheng, Q. Lan, M. Schnedler, P. Ebert, R. E. Dunin-Borkowski, "Counting point defects at nanoparticle surfaces by electron holography." *Nano. Lett.* **2022**, *22*, 6936–6941.

[33] F. Zheng, M. Beleggia, V. Migunov, G. Pozzi, R. E. Dunin-Borkowski, "Electron-beam-induced charging of an Al_2O_3 nanotip studied using off-axis electron holography." *Ultramicroscopy* **2022**, *241*, 113593.

[34] C. Gatel, A. Lubk, G. Pozzi, E. Snoeck, M. Hytch, "Counting elementary charges on nanoparticles by electron holography." *Phys. Rev. Lett.* **2013**, *111*, 025501.

[35] T. Suzuki, S. Aizawa, T. Tanigaki, K. Ota, T. Matsuda, A. Tonomura, "Improvement of the accuracy of phase observation by modification of phase-shifting electron holography." *Ultramicroscopy* **2012**, *118*, 21–25.

[36] R. Aso, H. Hojo, Y. Takahashi, T. Akashi, Y. Midoh, F. Ichihashi, H. Nakajima, T. Tamaoka, K. Yubuta, H. Nakanishi, H. Einaga, T. Tanigaki, H. Shinada, Y. Murakami, "Direct identification of the charge state in a single platinum nanoparticle on titanium oxide." *Science* **2022**, *378*, 202–206.

[37] C. Ophus, "Four-dimensional scanning transmission electron microscopy (4D-STEM): From scanning nanodiffraction to ptychography and beyond." *Microsc. Microanal.* **2019**, *25*, 563–582.

[38] M. J. Zachman, V. Fung, F. Polo-Garzon, S. Cao, J. Moon, Z. Huang, D. E. Jiang, Z. Wu, M. Chi, "Measuring and directing charge transfer in heterogenous catalysts." *Nat. Commun.* **2022**, *13*, 3253.

[39] Y. He, J. C. Liu, L. Luo, Y. G. Wang, J. Zhu, Y. Du, J. Li, S. X. Mao, C. Wang, "Size-dependent dynamic structures of supported gold nanoparticles in CO oxidation reaction condition." *Proc. Natl. Acad. Sci. U.S.A.* **2018**, *115*, 7700–7705.

[40] N. Ta, J. J. Liu, S. Chenna, P. A. Crozier, Y. Li, A. Chen, W. Shen, "Stabilized gold nanoparticles on ceria nanorods by strong interfacial anchoring." *J. Am. Chem. Soc.* **2012**, *134*, 20585–20588.

[41] H. Yoshida, Y. Kuwauchi, J. R. Jinschek, K. J. Sun, S. Tanaka, M. Kohyama, S. Shimada, M. Haruta, S. Takeda, "Visualizing gas molecules interacting with supported nanoparticulate catalysts at reaction conditions." *Science* **2012**, *335*, 317–319.

[42] T. Uchiyama, H. Yoshida, Y. Kuwauchi, S. Ichikawa, S. Shimada, M. Haruta, S. Takeda, "Systematic morphology changes of gold nanoparticles supported on CeO_2 during CO oxidation." *Angew. Chem. Int. Ed.* **2011**, *50*, 10157–10160.

[43] . Kuwauchi, S. Takeda, H. Yoshida, K. Sun, M. Haruta, H. Kohno, "Stepwise displacement of catalytically active gold nanoparticles on cerium oxide." *Nano Lett.* **2013**, *13*, 3073–3077.

[44] Y. Kuwauchi, H. Yoshida, T. Akita, M. Haruta, S. Takeda, "Intrinsic catalytic structure of gold nanoparticles supported on TiO_2." *Angew. Chem. Int. Ed.* **2012**, *51*, 7729–7733.

[45] S. Takeda, Y. Kuwauchi, H. Yoshida, "Environmental transmission electron microscopy for catalyst materials using a spherical aberration corrector." *Ultramicroscopy* **2015**, *151*, 178–190.

[46] T. W. Hansen, J. B. Wagner, "Environmental transmission electron microscopy in an aberration-corrected environment." *Microsc. Microanal.* **2012**, *18*, 684–690.

[47] H. G. Liao, D. Zherebetskyy, H. L. Xin, C. Czarnik, P. Ercius, H. Elmlund, M. Pan, L. W. Wang, H. M. Zheng, "Facet development during platinum nanocube growth." *Science* **2014**, *345*, 916–919.

[48] J. A. E. Hyllested, M. Beleggia, "Investigation of gas-electron interactions with electron holography." *Ultramicroscopy* **2021**, *221*, 113178.

[49] M. Schreiber, C. Cassidy, "Quantification of gas-based charge compensation by off-axis electron holography in open-cell environmental TEM." *Microsc. Microanal.* **2023**, *29*, 1575–1576.

[50] P. Haluai, M. R. McCartney, P. A. Crozier, "Detection of adsorbates induced changes on Pt/CeO_2 catalyst using in situ electron holography." *Microsc. Microanal.* **2022**, *28*, 1906–1907.

[51] S. Shimada, T. Takei, T. Akita, S. Takeda, M. Haruta, "Influence of the preparation methods for Pt/CeO_2 and Au/CeO_2 catalysts in CO oxidation." *Stud. Surf. Sci. Catal.* **2010**, *175*, 843–847.

[52] H. Lichte, M. Lehmann, "Electron holography–basics and applications." *Rep. Prog. Phys.* **2008**, *71*, 016102.

[53] Y. Midoh, K. Nakamae, "Accuracy improvement of phase estimation in electron holography using noise reduction methods." *Microscopy* **2020**, *69*, 123–131.

[54] T. Bar, T. V. de Bocarme, B. E. Nieuwenhuys, N. Kruse, "CO oxidation on gold surfaces studied on the atomic scale." *Catal. Lett.* **2001**, *74*, 127–131.

[55] R. Aso, Y. Ogawa, T. Tamaoka, H. Yoshida, S. Takeda, "Visualizing progressive atomic change in the metal surface structure made by ultrafast electronic interactions in an ambient environment." *Angew. Chem. Int. Ed.* **2019**, *58*, 16028–16032.

[56] A. Pfau, K. D. Schierbaum, "The electronic structure of stoichiometric and reduced CeO_2 surfaces: an XPS,

UPS and HREELS study." *Surf. Sci.* **1994**, *321*, 71–80.

[57] D. Widmann, R. J. Behm, "Activation of molecular oxygen and the nature of the active oxygen species for CO oxidation on oxide supported Au catalysts." *Acc. Chem. Res.* **2014**, *47*, 740–749.

[58] A. Tonomura, L. F. Allard, G. Pozzi, D. C. Joy, Y. A. Ono, *Electron holography.* Elsevier, Amsterdam, **1995**.

[59] P. E. Blochl, "Projector augmented-wave method." *Phys. Rev. B* **1994**, *50*, 17953–17979.

[60] G. Kresse, J. Furthmuller, "Efficient iterative schemes for *ab initio* total-energy calculations using a plane-wave basis set." *Phys. Rev. B* **1996**, *54*, 11169–11186.

[61] J. P. Perdew, A. Ruzsinszky, G. I. Csonka, O. A. Vydrov, G. E. Scuseria, L. A. Constantin, X. Zhou, K. Burke, "Restoring the density-gradient expansion for exchange in solids and surfaces." *Phys. Rev. Lett.* **2008**, *100*, 136406.

[62] S. L. Dudarev, G. A. Botton, S. Y. Savrasov, C. J. Humphreys, A. P. Sutton, "Electron-energy-loss spectra and the structural stability of nickel oxide: An LSDA+U study." *Phys. Rev. B* **1998**, *57*, 1505–1509.

[63] R. F. W. Bader, "Atoms in molecules." *Acc. Chem. Res.* **1985**, *18*, 9–15.

[64] G. Henkelman, A. Arnaldsson, H. Jónsson, "A fast and robust algorithm for Bader decomposition of charge density." *Comput. Mater. Sci.* **2006**, *36*, 354–360.
